# Capillary Hemangioma of the Middle Ear: One Case Report and Review of the Literature

**DOI:** 10.1155/2012/305172

**Published:** 2012-05-27

**Authors:** H. Nouri, A. Harkani, M. Elouali Idrissi, Y. Rochdi, L. Aderdour, A. Oussehal, A. Raji

**Affiliations:** ^1^Department of ENT, CHU Med VI, Marrakech 2360, Morocco; ^2^Department of Radiology, CHU Med VI, Marrakech 2360, Morocco

## Abstract

Hemangiomas are rare benign vascular tumors; there are several types including the capillary hemangiomas, we present the case of an exceptional localization of capillary hemangioma in the middle ear. We report the case of a 60-year-old female which consults for episodes of pulsatile tinnitus, otorragies, and hearing loss of the left ear. The clinical examination revealed a reddish polypoid mass in the left external auditory canal, the radiological tests showed a vascular mass in the middle left ear. The tumor was surgically removed, the histological study revealed a capillary hemangioma. The incidence of hemangiomas in the temporal bone, especially in the middle ear, is exceptional. Medical imaging guides to the vascular nature of these tumors that make confusion with other vascular tumors such as tympanic paragangliomas. The management is often surgical and the final diagnosis is histological.

## 1. Introduction

Hemangiomas are rare benign tumors growing in soft tissues and bones, there are several types of hemangiomas including the capillary type, hemangiomas are relatively common in head and neck, but they are exceptionally reported in middle ear [1, 2].

We report the case of a capillary hemangioma of the middle ear, through which we analyze clinical, radiological, and therapeutic aspects of this entity exceptionally encountered by the otologist.

## 2. Case Report

**Figure 1:**
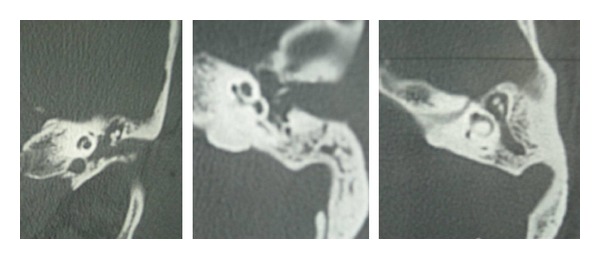
CT in axial and coronal, showing filling of the left middle ear without erosion of its bony walls or ossicles lesions.

**Figure 2:**
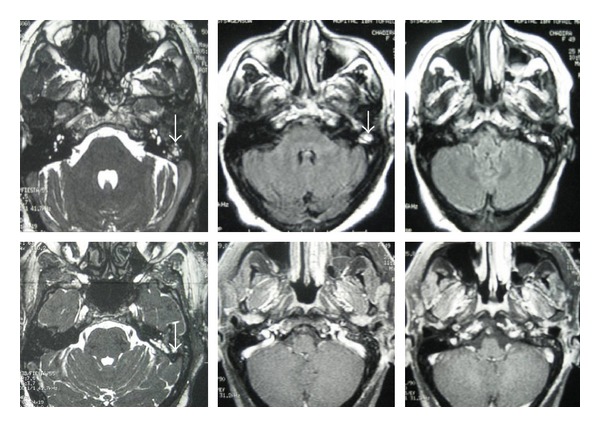
MRI showing a nodular lesion in intermediate signal T1 and Hyper signal T2 with enhancement after gadolinium injection.

We report a 60-year woman which presents, for six months ago, pulsatile tinnitus, hearing loss, and episodes of otorragies in the left ear. The otoscopic examination of the left ear revealed a reddish polypoid mass, sitting at the bottom of the external auditory canal and covered by a thin and inflammatory tympanic membrane. The examination of the right ear was unremarkable. The tonal audiogram showed a left conductive hearing loss of 30 dB, the audiogram of the right ear was normal. CT of petrous bone showed filling of the tympanic cavity without erosion of its walls and preservation of the ossicles (Figure 1). The MRI showed a nodular lesion, which appeared as an intermediate signal T1 and hypersignal T2 image, this lesion enhanced strongly after injection of gadolinium (Figure 2).

**Figure 3:**
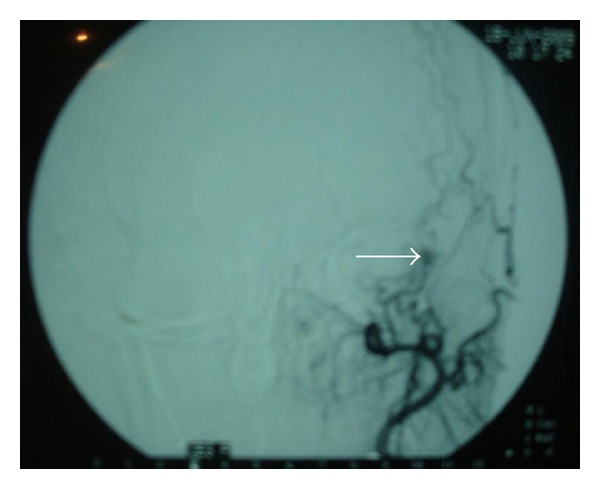
arteriography showing a “vascular blush” image.

The diagnosis of tympanic paraganglioma was suspected, in order to achieve a vascular mapping of this formation, an arteriography was performed and confirmed the vascular nature of the mass by giving a “vascular blush” image without being able to give a correct idea concerning the nurturing pedicle of this vascular formation (Figure 3).

The management of this case was surgical; after retroauricular incision, tympanomeatal flap detachment, and tympanic cavity entry, we discover a reddish bleeding mass occupying almost the entire tympanic cavity. In order to obtain better exposure of the mass and the ossicles, an enlargement of the auditory canal was performed by milling the posteroinferior wall of the canal.

The tumor was carefully peeled, using bipolar coagulation and fragmentation; it measures 1.5 cm in diameter and with intimate contact with the ossicles. The nurturing pedicle was provided by the tympanic artery.

The histological study of the mass concluded that it was a capillary hemangioma of the middle ear.

The postoperative period was uneventful; the evolution was marked by the disappearance of tinnitus and otorragies and also an auditory gain of 10 dB, there was no recurrences after 12 months of surveillance.

## 3. Discussion

Hemangiomas or angioendotheliomas are benign vascular tumors; they represent less than 0, 21% of all temporal bone tumors [1]. The temporal bone's hamngiomas have a predilection in three sites, which are by decreasing order of frequency, the geniculate ganglion, the internal auditory meatus, and the origin of the chorda tymapni [2, 3], we can explain this predilection by the vascular abundance in these sites [3, 4].

Middle ear localization of these tumors is extremely rare, the tumor is usually unilateral, and symptoms can vary from asymptomatic mass to noisy tumors with pulsatile tinnitus, otorragies, hearing loss, vertigo, recurrent middle ear otitis, or facial palsy [2, 3, 5].

The otoscopic examination shows usually reddish retro tympanic mass, more rarely; we can find a polypoid fleshy mass of the external auditory canal. The principal deferential diagnosis is the para ganglioma of the middle ear [3, 5].

At CT, the hemangiomas have the same density than the cerebral parenchyma and can sometimes, contain ossifications. At MRI, these tumors appear as a moderate or intermediate T1 signal and as a hypersignal T2. The arteriography sowed “vascular blush” image similar to glomus tumors [3, 5].

Histologically, the capillary hemangioma, the case of our patient, is characterized by well-differentiated vessels lined by a single layer of endothelial cells [1].

The treatment of theses tumors is essential because of their destructive and hemorrhagic potential.

The management is based on surgery which aims the complete resection of the tumor; indeed the recurrence rate, relatively high (16 to 23, 3% for nasosinusal localizations), depends directly on the quality of surgical resection [3]. Many cases with spontaneous regression were reported [4].

The surgical technique depends on the tumor seizes, the degree of hearing loss, and the situation of the jugular bulb [3, 6, 7].

The CO_2_ laser can be an interesting alternative to the conventional surgery; it allows better visualization of middle ear structures by reducing the bleeding [4, 5, 8].

## 4. Conclusion

The capillary hemangiomas of middle ear are extremely rare, the clinical and radiological features make confusion with other vascular tumors especially with the paragangliomas of middle ear. The definitive diagnosis is histological, and the management is based on surgery, the followup is capital because of the risk of recurrences.

## References

[1] Pavamani SP, Surendrababu NRS, Ram TS, Thomas M, Viswanathan PN, Viswanathan FR (2007). Capillary haemangioma involving the middle and external ear: radiotherapy as a treatment method. *Australasian Radiology*.

[2] Kojima H, Yaguchi Y, Moriyama H (2008). Middle ear hemangiona: a case report. *Auris Nasus Larynx*.

[3] Hecht DA, Jackson CG, Grundfast KM (2001). Management of middle ear hemangiomas. *American Journal of Otolaryngology*.

[4] Alobid I, Gastón F, Morello A, Menndez LM, Bentez P (2002). Cavernous haemangioma of the internal auditory canal. *Acta Oto-Laryngologica*.

[5] Hsueh PJ, Chen WY, Chiang YC, Lee FP (2007). Capillary hemangioma of the middle ear. *Otolaryngology*.

[6] Kostrzewa JP, Bowman MK, Woolley AL (2010). Middle ear hemangioma: a novel treatment for a rare problem. *International Journal of Pediatric Otorhinolaryngology Extra*.

[7] Rutherford KD, Leonard G (2010). Hemangiomas of the external auditory canal. *American Journal of Otolaryngology*.

[8] Álvarez-Buylla Blanco M, Vázquez Barro JC, López Amado M, Santiago Freijanes MP, Martínez Vidal J (2011). Capillary hemangioma of the middle ear: a case report. *Acta Otorrinolaringologica Espanola*.

